# Drug Rash With Eosinophilia and Systemic Symptoms (DRESS) Syndrome Due to Vancomycin

**DOI:** 10.7759/cureus.26219

**Published:** 2022-06-22

**Authors:** Mugdha P Kulkarni, Siddharth Chinta, Franklin Sosa, Rabih Nasr, Paul Kelly

**Affiliations:** 1 Internal Medicine, BronxCare Health System, New York, USA; 2 Medicine, BronxCare Health System, New York, USA; 3 Nephrology, BronxCare Health System, New York, USA; 4 Infectious Disease, BronxCare Health System, New York, USA

**Keywords:** eosinophilia, regiscar, hypersensitivity, vancomycin, dress

## Abstract

Drug rash with eosinophilia and systemic symptoms (DRESS) syndrome is a severe adverse drug reaction characterized by skin rash, fever, lymph node enlargement, and single or multiple organ involvement. Prompt diagnosis and withdrawal of the offending drug reduce mortality.

We report a case of DRESS syndrome along with a review of the literature. We identified the case as DRESS syndrome based on the skin rash, fever, eosinophilia, and liver and kidney involvement. According to the European Registry of Severe Cutaneous Adverse Reactions to Drugs and Collection of Biological Samples (RegiSCAR), our patient had a score of 6.

Drug rash with eosinophilia and systemic symptoms syndrome is a severe form of drug reaction with the potential for significant morbidity and mortality. Human leukocyte antigens (HLA) screening may be performed to prevent disease in susceptible patients. Steroids in a tapering dose are helpful in the resolution of symptoms.

## Introduction

Drug rash with eosinophilia and systemic symptoms (DRESS) syndrome is a severe form of drug hypersensitivity reaction that poses significant morbidity, and mortality with a death rate of approximately 10% [1], with the potential for long-term sequelae [2]. Typically the onset of symptoms is within three to eight weeks of initiating the culprit agent [2]. Common medications associated with DRESS syndrome include anticonvulsants, antimicrobials (most commonly the sulphonamides), lamotrigine, allopurinol, captopril, and antiretroviral drugs to name a few. Here, we describe a case of DRESS syndrome due to vancomycin.

## Case presentation

A 36-year-old male with a history of hypertension (HTN) and end-stage renal disease (ESRD) on hemodialysis (HD) via permcath presented to the emergency room with complaints of fever (1030F) and dyspnea. The physical examination was unremarkable. Laboratory results revealed pancytopenia with a total leukocyte count of 2.6(k/ul), platelets 145(k/ul), and RBC 2.94 (million/ul ) with hemoglobin 8.2 (g/dl). The patient had hyperkalemia with serum potassium 5.3mEq and serum creatinine 11.1mg/dl consistent with ESRD (Table 1). The chest X-ray revealed patchy bilateral infiltrates. He was started on empiric antibiotics for pneumonia with vancomycin, piperacillin/tazobactam, and azithromycin. 

**Table 1:** Comparison of lab parameters

Persistent Labs	Day of Admission	DAY 15
Hemoglobin (g/dl)	8.2	7.8
Platelets (k/ul)	145	133
WBC (k/ul)	2.6	13.4
Eosinophil %	0.00	18.4

**Figure 1:**
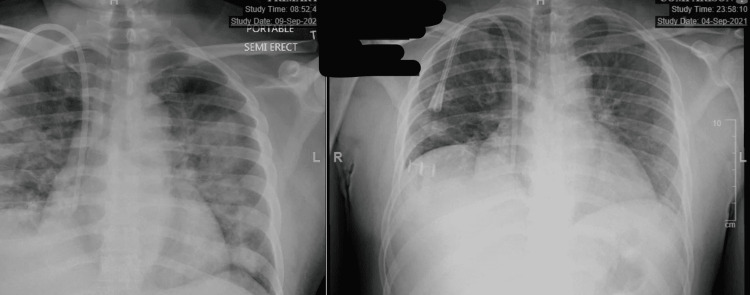
On the left is a chest X-ray done on day four of admission showing a worsening of the lungs. And on the right is the chest X-ray on the day of admission.

​​​​​The patient continued to be febrile on this antibiotic regimen. A repeat X-ray on day four showed worsening infiltrates (Figure 1). An infectious disease (ID) consult was obtained, and the patient was started on meropenem with doxycycline while continuing vancomycin. Blood cultures were negative. The patient was transferred to the intensive care unit (ICU) on day five due to hypoxia requiring bilevel positive airway pressure (BiPAP), tachycardia, and tachypnea. The patient's hypoxia, WBC, and platelet count improved on broad-spectrum antibiotics along with his clinical symptoms, and hence, he was transferred back to the medicine floor on day six. On day 15, the patient was noted to have a fever with a generalized macular rash on the extremities, back, upper chest, and more prominently on the face. A dermatology consult was called and recommended to rule out HIV and possible viral exanthem. The HIV was ruled out via serologic testing. In addition to the rash, labs revealed leukocytosis (total leukocyte count (TLC) 13.8) with eosinophilia and transaminitis. An ID specialist was re-consulted on day 16. Considering fever, leukocytosis with eosinophilia accompanied by typical rash after clinical improvement from bacterial pneumonia, negative blood cultures, and a negative HIV test, a diagnosis of DRESS was made.

**Figure 2:**
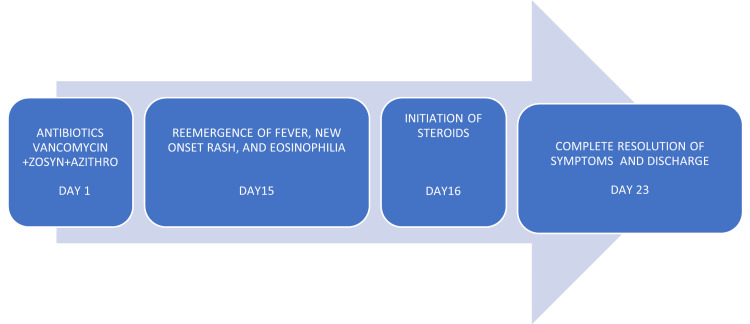
Course of events Image created by the author, Dr.Kulkarni.

Antibiotics were stopped and the patient was started on oral prednisone. The patient started showing improvement in his rash and became afebrile rapidly after starting corticosteroids and had complete resolution of leukocytosis, eosinophilia, and transaminitis. The culprit drugs suspected to be the cause of DRESS syndrome were beta-lactams, vancomycin, and less likely, hydralazine and/or clonazepam which were established medications the patient had been taking. On outpatient follow-up, the patient was clinically better with the resolution of the rash 12 days after initiation of corticosteroids (see Figure 2).

## Discussion

Drug-induced hypersensitivity syndrome (DIHS) is a systemic drug reaction also known as DRESS syndrome. It is an idiosyncratic reaction to medications. The condition is thought to be caused by an alteration in drug metabolism.

Drug rash with eosinophilia and systemic symptoms is a type IV hypersensitivity reaction [3]. Activated T lymphocytes release interleukin-5 (IL-5), leading to characteristic eosinophilia [4]. It may reactivate latent viruses such as human herpesviruses, Epstein-Barr virus (EBV), and cytomegalovirus. Since certain human leukocyte antigen (HLA) subtypes can be associated with certain drug susceptibilities, HLA subtype analysis can help identify patients at risk [3].

The diagnosis is based on clinical and laboratory findings. Typical presenting signs and symptoms include fever, facial swelling, lymphadenopathy, and morbilliform rash with end-organ involvement including hepatic, renal, pulmonary, hematologic, cardiac, and endocrine abnormalities [5]. The laboratory findings include leukocytosis with eosinophilia or atypical lymphocytosis, transaminitis, hepatitis, increased serum creatinine, and pyuria indicating nephritis. Eosinophilia is not always present hence the term DHIS [6]. Our patient had a fever, rash, leukocytosis with eosinophilia, and liver involvement that manifested as transaminitis.

The cutaneous reaction usually begins two to eight weeks after the drug is started and persists even after drug cessation [6]. In our case, a rash appeared 14 days after starting antibiotics. The liver is the most common organ involved in almost 50% of cases, followed by renal involvement (10%) while pericarditis, myocarditis, and pneumonitis are less common. With visceral involvement, there is a 10% mortality risk [4]. Patients should undergo cardiac evaluation in suspected as well as severe cases with help of an electrocardiogram and echocardiogram.

The most common drugs associated with DRESS syndrome are anticonvulsants phenytoin, carbamazepine, and lamotrigine [7]. Other drugs include allopurinol, antibiotics such as sulfonamides, cephalosporin, and penicillins. In The European Registry of Severe Cutaneous Adverse Reactions to Drugs and Collection of Biological Samples (RegiSCAR) study, clear drug causality was found in 88% of DRESS while in 2% etiology was unclear [6-8]. In our patient, DRESS syndrome developed with vancomycin as the most possible cause. Other drugs like hydralazine and clonazepam were also suspected to be the possible inciting agents but vancomycin seems more plausible given the time frame of symptoms. Piperacillin/tazobactam was switched to meropenem after three days of its initiation.

Vancomycin is accountable for about 2/3 of the antibiotic-associated DRESS syndrome [9]. In an electronic health record study, 74% of DRESS cases were associated with antibiotics out of which most cases were attributed to vancomycin followed by beta-lactams [10]. Our patient received only three days on piperacillin/tazobactam whereas vancomycin was continued until day 13. Hence, we believe that vancomycin is the causative agent for DRESS syndrome in our case though piperacillin/tazobactam cannot be excluded with certainty. Meropenem was started on day four of hospitalization and the duration from its initiation to the onset of rash is 10 days which makes it highly unlikely to be the causative agent. The diagnosis of DRESS is challenging with many cases initially misdiagnosed as an infection. The register of severe cutaneous adverse reactions (RegiSCAR ) created a diagnostic validation score that is used for the diagnosis [11]. The criteria are scored from 4 to 9, with scores of less than 2 excluding the diagnosis of DRESS, 2 to 3 being possible, 4 to 5 probable, and score >5 being definite. The RegiSCAR score for our case was 6.

The management includes prompt cessation of causative medication. Identification of the culprit drug can be difficult especially when multiple medications have been administered. Patients usually need hospitalization with those in shock requiring intensive care. A dose of 1mg/kg of oral prednisone or its equivalent is recommended as a starting dose. Steroids are tapered gradually over three to six months after laboratory normalization [12]. In case of recurrence after steroid taper, mycophenolate mofetil can be used [6]. Outpatient follow-up for ensuring resolution of systemic involvement and monitoring for reactivation of latent viruses should be done. Development of autoimmune diseases such as thyroiditis and diabetes have been reported. Hence adequate follow-up of patients is necessary [3,6]. Our patient showed significant improvement both clinically and in laboratory trends as well as symptoms with steroids. Hence patient was discharged with outpatient follow-up to confirm resolution and was tapered off steroids.

## Conclusions

Drug rash with eosinophilia and systemic symptoms syndrome is a severe form of drug hypersensitivity. Common differential diagnoses include Stevens-Johnson syndrome, toxic epidermal necrolysis, hypereosinophilic syndrome, Kawasaki disease, and Still's disease. Minimum laboratory tests like complete blood cell count, liver function tests, serum creatinine level, and urine analysis help to differentiate DRESS syndrome from other causes and to identify asymptomatic internal organ involvement. Diagnosis of DRESS is often delayed leading to complications and even death. Prompt diagnosis and timely treatment reduce morbidity and mortality significantly. The mainstay of treatment is stopping the offending drug with the addition of topical or systemic corticosteroids. Vancomycin is one of the antibiotics implicated in precipitating DRESS syndrome, the others being minocycline, sulfonamides, beta-lactams, cephalosporins, piperacillin-tazobactam, and fluoroquinolones. A follow-up to ensure the resolution of symptoms is recommended for all patients. Also, well-designed clinical trials are necessary for improving the management of this rare syndrome.
